# The development process of a type 2 diabetes health-promoting CBPR intervention

**DOI:** 10.3389/fpubh.2025.1486996

**Published:** 2025-01-31

**Authors:** Cecilia Lindsjö, Katarina Sjögren Forss, Christine Kumlien, Anders Kottorp, Margareta Rämgård

**Affiliations:** ^1^Department of Care Science, Faculty of Health and Society, Malmö University, Malmö, Sweden; ^2^Research Centre Promotion for citizen health, Malmö University, Malmö, Sweden

**Keywords:** type 2 diabetes, health literacy, peer support, community-based participatory research, migration, women, health promotion

## Abstract

**Introduction:**

Participation is one of the core elements of health promotion, which means that approaches and methods should focus on involvement. The process of involving women with a migration background in health promotion needs to be further explored. Thus, the aim of this study was to explore the development process of a type 2 diabetes health-promotive community-based participatory research intervention among Middle Eastern women with a migration background, living in Sweden.

**Materials and methods:**

This study was performed within the context of a community-based participatory research program in Sweden. The design of this study followed the development process of a community-based participatory research conceptual model, including three of the original four dimensions, that is, the context, the partnership process, and the intervention and research dimension. Appropriate methods for data collection were used in the various dimensions. Participants from the community, active in the program, conducted dialogue cafés, together with the core partners of the program, to inventory existing needs as well as what actions were needed for promoting health and thereby prevent type 2 diabetes.

**Results:**

The two dialogue cafes resulted in one long term and three short term goals. The third short-term goal—create health circles around food and nutrition was decided to be in focus for this study together with cooperation with the local health care center. The partnership process made it possible to involve relevant collaborators, which resulted in a jointly developed nurse-led educational intervention. Participants and stakeholders were also involved in the process of modifying and elaborating evaluation tools appropriate for the intervention.

**Discussion/conclusions:**

The community-based participatory research approach enables the acknowledgement and use of the various kinds of knowledge of all stakeholders, including the community members. In this study, the community members’ knowledge was obtained through participation and dialogue, aimed at balancing power between stakeholders. This approach, that is, developing a community-based participatory research intervention, offers a possibility for the primary health care to engage with the community members and for other stakeholders to work in a health-promotive way.

## Introduction

A trustful relationship in health care has been described as important for building health equity and improving integration (1). And the participatory approach entails an opportunity for partners to collaborate for migrant health (2). This may be vital since previous research conveys that people in Sweden with a migration background from the Middle East have twice the prevalence of type 2 diabetes (T2D) compared to native Swedes (3). Also, treatment control of T2D is poor among migrants, potentially increasing the risk of complications of the disease (4). Thus, migrants from the Middle Eastern region have been identified as a group to be focused upon for health-promotive interventions regarding T2D (4). A combination of diet and physical activity promotion programs can be used to reduce the incidence of T2D among people with increased risk of T2D (4). In addition, a culturally adapted lifestyle intervention has shown to influence the metabolic profile positively, and thus the risk of developing T2D, among Iraqi migrants at risk of developing T2D (5). However, it seems that the effect of such lifestyle interventions is smaller among migrants compared to native Europeans, and results are scarce regarding how to deliver lifestyle interventions among migrants to prevent the development of T2D (4). A good collaboration between community and community actors has previously been acknowledged in a study aimed at promoting health and preventing T2D in a disadvantaged area in Sweden (6). Furthermore, MacFarlane et al. emphasize that migrants should be involved in decision-making regarding health and conclude that a participatory approach is well suited (7). Additionally, a previous Norwegian study highlighted that intervention studies and participatory approaches may contribute to migrant health research and to greater competence around health issues within this population (8). However, in a scoping review it was concluded that the reporting of participatory health research among women with a migration background could be enhanced by highlighting the process of how the involvement with the community has been handled (9), which may include how the community’s knowledge has been taken into account.

“Health promotion is about creating and strengthening conditions for health, regardless of the presence of disease or not” (10) (p.28) (our translation), and participation is one of the core principles of this approach (11). A participatory approach means that the perspective and the knowledge of the ones that the research is about, are acknowledged and valued as much as other ways of knowing and thus seen as a resource in the research (2). Intersectoral work, that is, collaboration between various relevant operational stakeholders in the society, is another core principle of health promotion work (11). This supports a further core principle, namely, sustainability, since involving relevant partners from the local community makes it possible for the knowledge and competence gained to stay within the community when interventions withdraw. The approach of community-based participatory research (CBPR) offers a process whereby partners can work collaboratively together with the community (12). CBPR is rooted in the same tradition as other participatory approaches, such as participatory action research (PAR) and participatory health research (12). Research concerns should be context bound and spring from the local community for optimal relevance (12). However, power structures from different stakeholders may permeate populations’ feelings regarding their right to speak and may be disempowering (13). Therefore, breaking down hierarchies is a key element of the approach. Paulo Freire worked with and coined the concept authentic dialogue (13, 14). Dialogue can be seen as a tool for participatory practice, where dialogue is central in order to share and listen to each other’s perspectives (13). Dialogue includes interaction between two or several parties and has the potential to open up for critical consciousness (13). It is the critical consciousness that the participatory research tradition strives to realize as a pathway to democratization and emancipation for social change (15). The creation of communicative spaces to enable dialogue is therefore a task that needs to be handled, and that concerns both time and physical place, as well as what can be referred to as a space for change (16). The physical place needs to be a meeting point where everyone feels welcome (17). The collaboration and dialogue bring about the ability to acknowledge and benefit from all partners’ various knowledges to reach the common goal (12). All types of knowledge can then be produced—not only propositional knowledge, which is usually what is produced in traditional science approaches (18), but also experiential, presentational, and practical knowledge that can be produced using a cycle of action and reflection, which is the research cycle of action research (19). Emotional and intuitive ways of knowing, as well as knowledge coming from critical reflexivity and consciousness, are highlighted, and methods generating that kind of knowledge are preferred (13). In a way to counteract power structures, one should take the chance to reinforce one’s own listening, so that one actually hears when engaging with others in dialogue (13), to also hear the tacit knowledge (20). To hear is here not only about hearing physically but also about having a willingness to transform oneself by what one hears (21). Truly listening, and being serious about what one hears, is a way of being respectful and of acknowledging each other’s equality (13). Freire asserted that it is everyone’s right to be involved in dialogue, and that belief brings about a belief in others, which is the base for dialogue and for creating trust (14). In such a way one can reach a horizontal communication, and trust can be built, trust being at the core of a functioning equal partnership (13).

Dialogue methods that promote horizontal communication and trust are preferred in CBPR and participatory practice. Such practice provides tools for disrupting customs influenced by power structures, which can open up for the awareness of how power dynamics are directing our ways of living and choices in everyday life (13). Thus, this process includes reflection, and, consequently, the role of a researcher is also to promote the practice of reflection. Reflection is a process aimed at making sense of phenomena in the world and is based on critical thinking (13). It is essential for development to be able to change one’s own sense-making (13). In combination with dialogue, one can go from questioning everyday life phenomena to action for change together (13). It is through questioning that empowerment can be developed (13). The iterative process of reflection and action is also one of the core parts of the CBPR process, and something the partners should undertake in order to promote shared sense-making (12). This is what Freire called praxis, that is, reflective action (14). It is in dialogue, Freire claimed, that reflection and action unite for change (14).

When not acknowledging, for example, minority groups’ accounts of the world, knowledge production can be skewed in favor of privileged groups, a phenomenon known as epistemic injustice (22). By using participation and dialogue and creating a communicative space, we wish to enable reflection and action in this study. A communicative space may be important not only for community members, but also for other stakeholders that may—as Freire argues—need to raise their consciousness about their own privileged situation (14), which relates to what Fricker suggests as an epistemic goal: increasing the “testimonial sensibility” of the hearer (22). Thus, as the CBPR approach is based on and admits various sorts of knowledge (23) it may be a way of counteracting epistemic wrongs and injustices as well as health inequities. When not only acknowledging propositional knowledge but using instead resources to bring forward also other sorts of knowledge, persons’ testimonies can be given credibility (24). This may also go hand in hand with the new national reform that is implemented in Sweden, namely, “Nära vård” (Close Care), where the patients are meant to be more included in their own care and where the health care professionals are to work in a more health-promotive and collaborative way with other actors too (25). What is more, health promotion is closely linked to health literacy, and is therefore valuable to consider in health promotive work (11) (p. 41). And, Nutbeam and Lloyd emphasize that the number of health literacy interventions is low (26), and that the need of research on health literacy is more to do with how interventions can be accomplished in the real context of a clinical setting (26) as well as on how to targeting communicative and critical health literacy (C & CHL) among community populations (27). Thus, the aim of this study was to explore the process of developing a T2D health-promotive CBPR intervention among Middle Eastern women with a migration background, living in Sweden.

## Materials and methods

### Design

**Figure 1:**
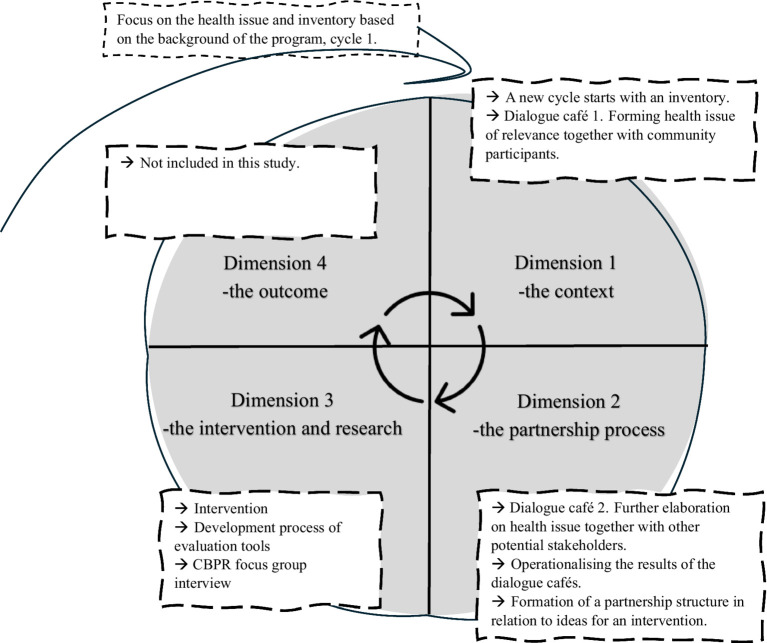
Dimensions of the development process according to the conceptual model of the CBPR intervention.

The design of the study follows the dimensions of a conceptual model of a CBPR intervention, developed by Wallerstein et al. and modified and used for planning within the program where this study is situated (Figure 1) (15). The model includes four dimensions: *the context, the partnership process, the intervention, and the outcome* (15), and it is a way for partners to work collaboratively, acknowledging dialogue and power perspectives while striving for health equity and social justice. *The context dimension* is about inventorying contextual factors in the local setting and resources, as well as prioritized health issues (15). It includes inventorying the history of collaborations, local policies, or decisions that may influence the starting project, and making sure the health issue in focus is a concern for the community (15). In the *partnership process dimension*, an exploration of partnership structures within the community is conducted, in order to build on local resources (15). *The intervention dimension* is where the intervention is developed, based on dimension one and two, and then the appropriate research methods are chosen, making sure this is conducted together with the community, including their knowledge and cultural norms (28). *The outcome dimension* can, for example, be research results concerning physical and mental health, but it may also pertain to health-related social issues, such as inclusion, empowerment, and trust (15).

### Context

The current study was conducted within the “Equal Health—Collaborative Innovations for Health Promotion” program in Lindängen, Malmö, Sweden, a CBPR program aiming to “create new ways to improve health through participatory and cooperative strategies in a community health promotion platform” (29) (p. 4). Malmö is the third-largest city in Sweden with approximately 360,000 inhabitants, and Lindängen is a socially deprived part of the city (30). This CBPR program has been ongoing since 2016. Besides academia and the local community, several other stakeholders are involved from the public, non-governmental, and private sectors in the society, and they all collaborate in a penta-helix model (30). The program was initially formed with a strategic group and a hub, that is, the program’s operative working group where various representatives of stakeholders participate. Using the penta-helix structure in the program, means that stakeholders from various sectors of the society are represented and that their voices are equally valued. This signifies that also members of the community are involved in the decision-making in the program’s processes on equal terms, so as to ensure that the community’s needs are fulfilled. When the first CBPR cycle started in 2016, five different labs were created: the women’s health lab, the oral health and food lab, the social health lab, the mental health lab, and the physical activity lab (29). The women’s health lab and the oral health and food lab focused on also promoting health for chronic diseases (17, 31).

This study is based on the second cycle of the program, which started during the autumn of 2022. The reason for starting the second cycle was to conduct an inventory to get an overview of community needs, activities, and stakeholders, for example, to find out whether the constellation of the partners within the program and activities had changed (32). It was decided that the new cycle should focus on T2D due to previous results of the program. For example, during planning meetings in the community in 2018/2019, it was highlighted that participants required more knowledge on diabetes and food (17). Thus, a nurse specialized in diabetes and a dietitian from a local health care center were invited as guests to health circle meetings in 2019 (17). Furthermore, in another study conducted within the program, diabetes was highlighted as a subject to focus upon since participants felt they had a poor understanding of their own health status regarding their diabetes diagnosis or the risk for it (33). They also felt uninformed by the health care regarding measurements warranting an examination for diabetes and wished to be examined more closely with regard to blood glucose (33). The focus on T2D was even more enhanced in 2020, when Malmö, the city where this program is located, joined Cities Changing Diabetes (CCD) (34). A collaboration started, where the municipality, the regional health care organization, Novo Nordisk, and Malmö University work together around the prevention of T2D (34). In the mapping phase in Malmö, it was revealed that T2D was associated with the socioeconomic circumstances of the population and that it was more common in the disadvantaged areas of the city, such as Lindängen (34, 35).

The need of a new inventory was identified by the local hub in Lindängen with representation from the local hub’s main organizations (academia, the community, non-governmental organizations, and the municipality) (30). A doctoral student (CL) was at the time visiting the local hub and was thus included in conducting the research. This local hub is the operational working team within the community that follows the full process of the community’s needs of activities from initiation to evaluation to new initiation, and takes action for planning for the health promotion activities (32). For this study, the hub can be described as a research team, facilitating the process (36).

**Figure 2:**
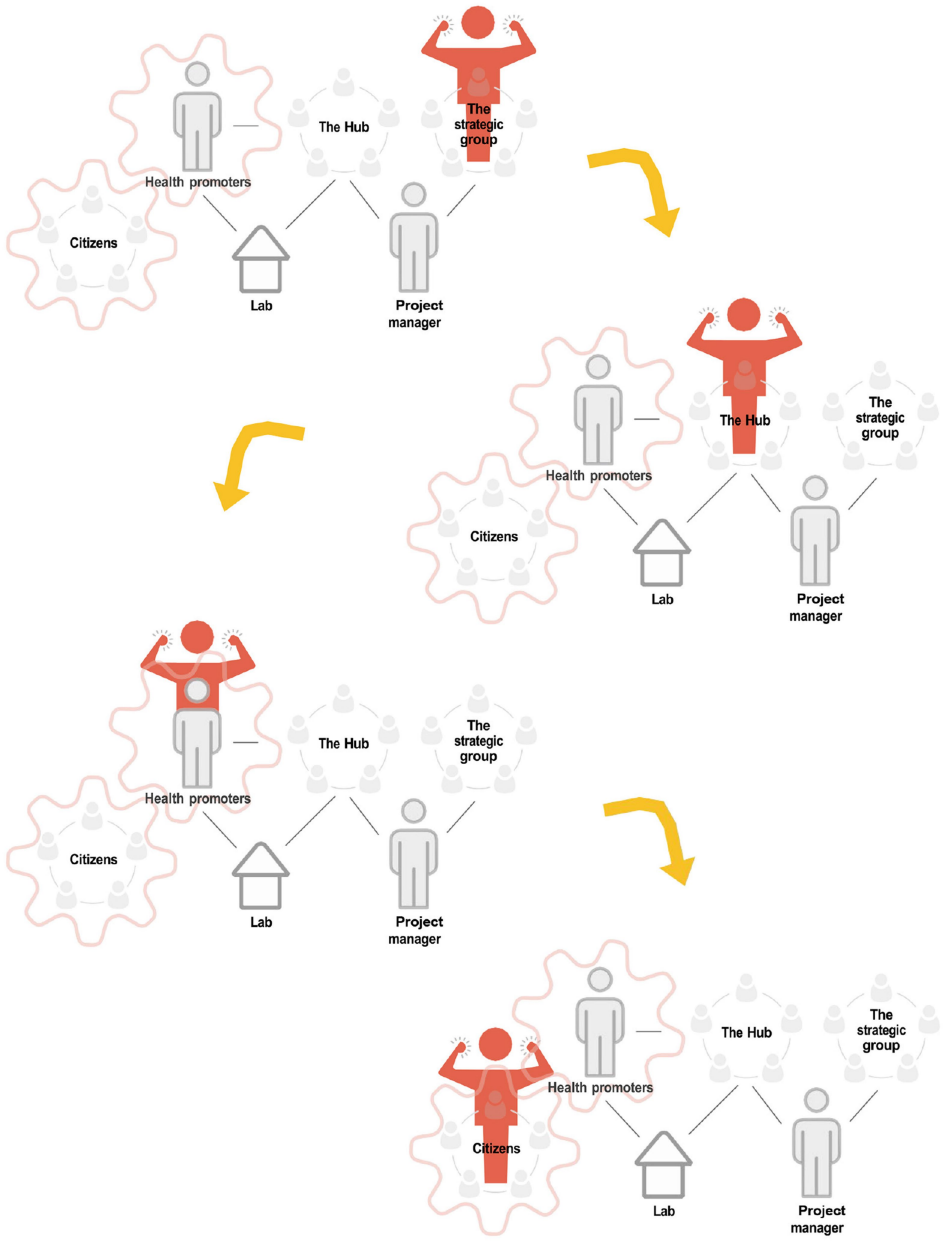
Working model in the collaborative innovations for health promotion program of how power should be transferred (32).

Employed lay health promoters (LHPs) facilitate the program’s health promotion activities together with the citizens, in social meeting places in Lindängen (37). The LHPs are also part of the strategic group of the program as well as the hub—the program’s operative working group, where they represent the community (32). They are working with health promotion activities, such as health circles and physical activities, in a participatory dialogue (17, 37). LHPs can be described as brokers and links between the community and the other partners of the program (37). They are bilingual and from the same community, Lindängen, as the participants, but they also have knowledge of the context outside the community. Their role is multifunctional, including trying to translate between perspectives, organizing activities, and coordinating contacts with participants, and also balancing between being a full member and an intruder in the groups where they operate (38). The LHPs are essential in the process of transferring the power from the strategic group to the community (Figure 2) and vital in the process to create communicative space. They are connected to the context of the place and in a position to build capacity in the community.

### Participants

The inventory initiative started with needs being identified in the two labs that were still active within the program—the women’s health lab and the oral health and food lab, each led by an LHP. The LHPs are in daily contact with around 20 women each from the community, through their activities, and many more women are connected to the labs. These two labs consist of women with a migration background, mainly from Middle Eastern countries, such as Iraq and Syria, but also from North Africa, and the labs are a result of the formation of the program in 2016, which has been described elsewhere (29). The age of the women spans between approximately 25 and 75 years, and the length of their time of residence in Sweden also differs, from just a few years to some decades. The length of education and their daily occupation vary, too, but most of them were not working, which is why day-time activities were suitable for them. Both women that had been connected to the labs for some time and new participants were invited. Due to previous participation within the program, a relationship of trust had already been established between stakeholders, the LHPs and the participants, and new participants were usually informed by peers. However, trust is an ongoing process that should not be overlooked (15).

### Data collection and analysis in the development process of intervention

#### Dialogue cafés

**Figure 3:**
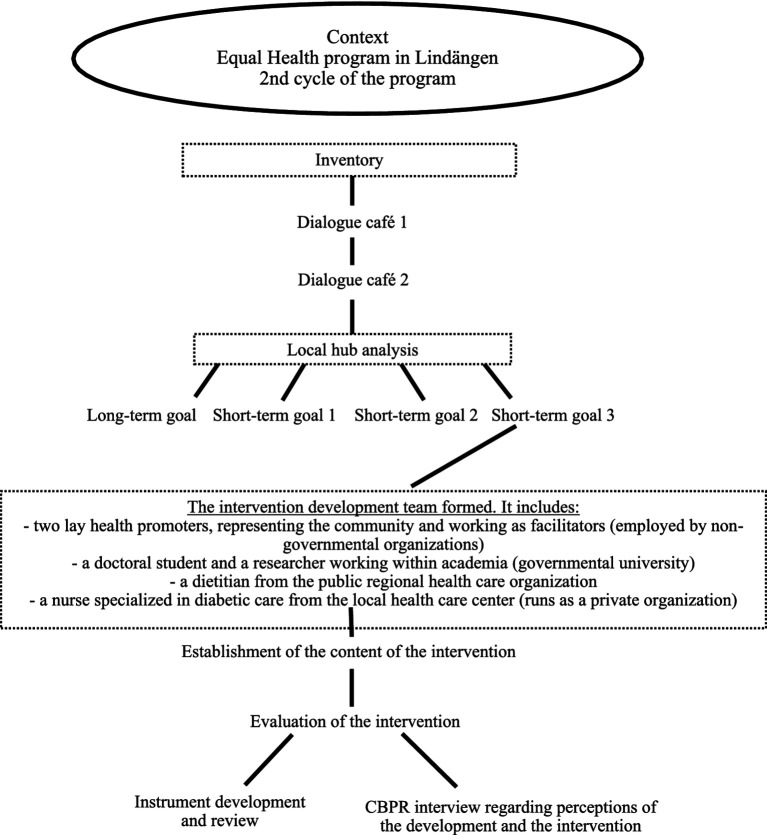
The process of the development of the CBPR-intervention.

The inventory of needs and possible actions, in relation to the prevention of T2D, was planned to be conducted with a first and second dialogue café in the autumn of 2022 (Figure 3). A version of world cafés was used as a method for providing communicative space (13). This is a dialogue method to be used when involving many people at the same time and where all need to be included in the dialogue. Thus, participants are grouped around smaller tables for separate dialogues (13). Both sessions were conducted in Arabic and Swedish, and thereby participants had a chance to express themselves in their mother tongue as well as getting the dialogue translated. Each session included group work, where each group worked through the questions in focus separately.

In the first dialogue café only participants from the women’s health lab and the oral health and food lab was invited, to give them interpretive priority in identifying the needs of health promotion. This was a strategy meant to handle the power balance, as they as a community were given a chance to identify their common needs together before other stakeholders were included in the process. The first dialogue café was conducted with the following focus: *Inventory of needs of health promotion activities to prevent diabetes*, and the questions to participants were: “*What do you think about when you hear the word ‘diabetes’?*,” “*What do we need to do to prevent the onset of this disease?*,” and “*What can we do by ourselves and what do we need help with?*.” The dialogue café was conducted in the meeting place where the women from the community usually met for activities, and which was thus familiar to them. The LHPs coordinated the session, with the help of women from the labs, as they knew the community and had the double language skills needed to handle the session, and so that the community themselves could manage the dialogues. All the women were directed to a table upon arrival. At each table, one facilitator from the community was allocated to keep track of the questions and was chosen to be the spokesperson for the group. Each group wrote their answers to the questions in the form of mind maps that was then used for a joint analysis.

The analysis of the first dialogue café was conducted by the participants and the hub as one part of the session. Thus, after the group work the analysis was conducted and joint mind maps with all the groups’ work were created around each question. The written conclusions of the questions from each group were categorized on the wall, and together themes were identified.

Then, in the second dialogue café, potential stakeholders were also invited, together with the women, to a new dialogue café. The second dialogue café was conducted based upon the themes arrived at by the women during the first dialogue café. Thus, the second dialogue café was a tool to spread the participants’ ideas about health promotion around T2D to the other stakeholders, in order to explore possibilities for actions. Potential stakeholders that could be valuable for the way forward were identified by the hub and members of the community, starting with those stakeholders that were already known because they had to some extent been involved in the program earlier, such as the local library and the oral health company TePe. Local stakeholders that were new to the program, for example, from the local health care center and the local supermarket, were also approached and invited, generally based on already known contacts; thus, recruitment was usually conducted by snowball sampling. For example, as the women had suggested more contact with the health care, the local health care center was invited to the second dialogue café. The LHPs were central in this work, since their work in the penta-helix model during the last years had resulted in a large network of stakeholders. Around 100 people announced their participation in the second dialogue café, and they were all divided into groups by the hub. The group allocation was based on their relation to each theme, according to their job position, resulting in 10 groups. Thus, as there were five themes, each theme was represented in two groups. Efforts were also made to allocate stakeholders in such a way that all sorts of sectors from the penta-helix model were represented in each group, that is, the community, academia, public organizations, private organizations, and non-governmental organizations, so as to maximize the number of different voices. Besides, at least two women from the community were usually allocated in each group to facilitate the dialogue and balance power by supporting each other. The second dialogue café was arranged with the following aim: *How can we together prevent type 2 diabetes and reach better health through health-promoting activities?* The hub prepared questions for the dialogue café to be worked through in each group, in relation to the group’s theme, and the work was structured thus: inventory the problem, identify needs and possible actions, and then prioritize the actions. The LHPs, together with volunteering participants from the labs as facilitators, were central coordinators of the second dialogue café, too, with support by the hub. After the group work of the second dialogue café, an oral presentation of each group’s work to all participating in the dialogue café was conducted. Data from the dialogue cafés were collected in the form of notes.

The material from the second dialogue café was analyzed by the hub inspired by thematic analysis, as described by Braun and Clark (39). It was first collated according to the main ideas that emerged, which were: community/health center, health circles, spreading of information, health care, food habits, physical activity, excursion, and, lastly, a mixed category. Then the local hub together discussed what activities were already in place and what ideas were new in the material, and they subsequently identified what actions needed to be taken and set priorities, including both long-term and short-term goals.

#### Partnership process and formation of the intervention

The result from the dialogue cafés was then to be implemented in the spring of 2023. Thus, the doctoral student, the LHPs, and a representative of academia within the hub started to form thoughts about an intervention and initiate contacts for the formation of partnership structures in relation to ideas for an intervention.

When the partnership and the intervention had been formed, the aim was to evaluate the intervention quantitatively. However, as the evaluation tools were to be processed and the ethical approving for those was going to take time it was decided, within the intervention facilitating team to test the intervention during March and April 2023 to participants of one of the labs facilitated by one of the LHPsc, without quantitative evaluation in this study.

#### CBPR focus group interview

For the participants to reflect about the intervention and development, a CBPR focus group interview (40) was conducted in the spring of 2023, during a test of the intervention was conducted. The interview guide used was based on the CBPR partnership guide developed by Wallerstein et al. (40). This is a tool to evaluate the partnership and includes domains such as involvement, context, and behavior in the group and partnership. Participants attending the intervention were invited to the interview and eight out of 20 accepted. The focus group interview was conducted after six out of 10 meetings, thus not after full intervention. It was performed in a place familiar to the participants, namely, their community meeting place where they meet several times a week. One of the researchers facilitated the dialogue, which was digitally recorded. It was conducted in Arabic and Swedish, and the LHP who coordinated the group translated the dialogue. The digitally recorded material from the CBPR focus group interview was listened through. Data that were related to the aim of the study and that contributed information about the study process, were extracted. These extracts were then analyzed inspired by thematic analysis, as described by Braun and Clark (39), by categorizing participants’ accounts.

### Data collection and analysis in the development process of the evaluation tools

#### Selection of evaluation tools

When the intervention had partly been formed in the spring of 2023, the researchers initiated the search for evaluation tools that could match the intervention and the population. As shown by previous results of the program, participants required measurements to feel more informed about diabetes and health status (33). Also, since weight loss and stress reduction were the overarching goals of the participants, according to the outcome of the dialogue cafés, it was relevant to keep track of the measurements related to those goals to enable better self-care. Therefore, it was suggested that weight, height, and waist circumference were to be measured. Furthermore, it was suggested that stress be measured by a Visual Analogue Scale (VAS).

Regarding the subject of food and food habits, a food frequency questionnaire was viewed as appropriate. After comparing different tools, a questionnaire previously used in a national health promotion intervention in a northern county in Sweden, was chosen. However, the questionnaire needed modifications to suit the study population, since it was extensive. The Food Frequency Questionnaire (FFQ2020), designed within the Northern Sweden Diet Database (41), was thus used in this study but with modifications to better match the target group.

Health literacy was also relevant as a focus of evaluation, according to the themes arrived at during the dialogue café and the requested mode of group activities, and also according to results from previous studies in the program. The Swedish Functional Health Literacy scale (S-FHL) (42) and the Swedish Communicative and Critical Health Literacy Scale (S-C & CHL) (43) were found applicable and appropriate for the health promotion evaluation.

Another aspect relevant for the evaluation was quality of life (QoL), since finding a balance in everyday life was identified as a goal to strive for among participants. Also, the improvement of QoL among participants is an objective of CBPR (12). Furthermore, QoL can be a relevant measure of health promotion interventions (44). Hence, the WHOQOL-BREF, which has been used previously in the program, was selected as an evaluation tool (45).

It was also valuable to be able to describe the study population, and therefore a study population demographics tool was formed, including characteristics such as age, country of birth, years residing in Sweden, and educational background. As that information was gathered from the participants, it was crucial that the questions and categories were clear and easy to understand.

The intervention facilitating team suggested the above evaluation tools, which were then presented to a group of participants from the women’s health lab, who were invited to think about and reflect around their appropriateness, as part of the decision-making process.

#### Cognitive group interviews

As part of dimension three, related to research, evaluation tools were modified and developed for the intervention. The participants’ perspective on and thoughts about the evaluation tools were important for further development. Thus, to test the validity of some of the instruments in a relevant study population, a modified version of qualitative cognitive group interviews was conducted together with the women, and the interviews were digitally recorded (46). The method was modified because of language limitations; the interviews needed to be translated and to be completed as group interviews. The aim of testing the questionnaires was to identify any issues, detect the representativeness of the items for the participants, and discover possible usability difficulties for participants. The evaluation tools were therefore filled out in groups and participants were encouraged to ask and comment when having difficulties understanding, filling out the tool or to highlighting other issues. One of the interviews included five participants and the other interview included seven participants. In both interviews, the doctoral student served as the facilitator and one of the LHPs translated, and in one of the interviews another researcher observed and took notes.

Data from the digitally recorded cognitive interviews were extracted, compiled, and categorized, based on the participants’ suggestions of modifications. Also, material from notes taken during the sessions were extracted and categorized. This was an inductive procedure where any kind of suggestions were extracted from the material. Those were compiled and then categories were identified.

## Ethical considerations

Within participatory research, ethical challenges have previously been noted in the collaboration between partners, or in the ownership of the findings, as well as in the ethical application process (47). The CBPR approach means that interventions or data collection instruments are not pre-decided by the researchers and, consequently, that information cannot be included in an ethical application before the research takes place. However, in this study an ethical approval for the communicative spaces and its findings were in place when the second cycle started (Regional Ethical Committee in Lund, Reg. no. 2018/591). When the intervention was more developed and data collection methods and instruments were decided on, a supplementary application was processed and approved. Participants were given information about the research, orally in their mother tongue and in writing in Swedish, prior to the data collection. Written consent was also collected from the participants.

## Results

The result encompasses the development process of a CBPR intervention as well as modifications of the evaluation tools. Thus, the process corresponds to dimensions one, two, and three of the CBPR intervention conceptual model: the context, the partnership process, and the intervention and the related research to the CBPR intervention conceptual model.

### Development process of the CBPR intervention

#### Identifying goals for an intervention based on dialogue café results

**Figure 4:**
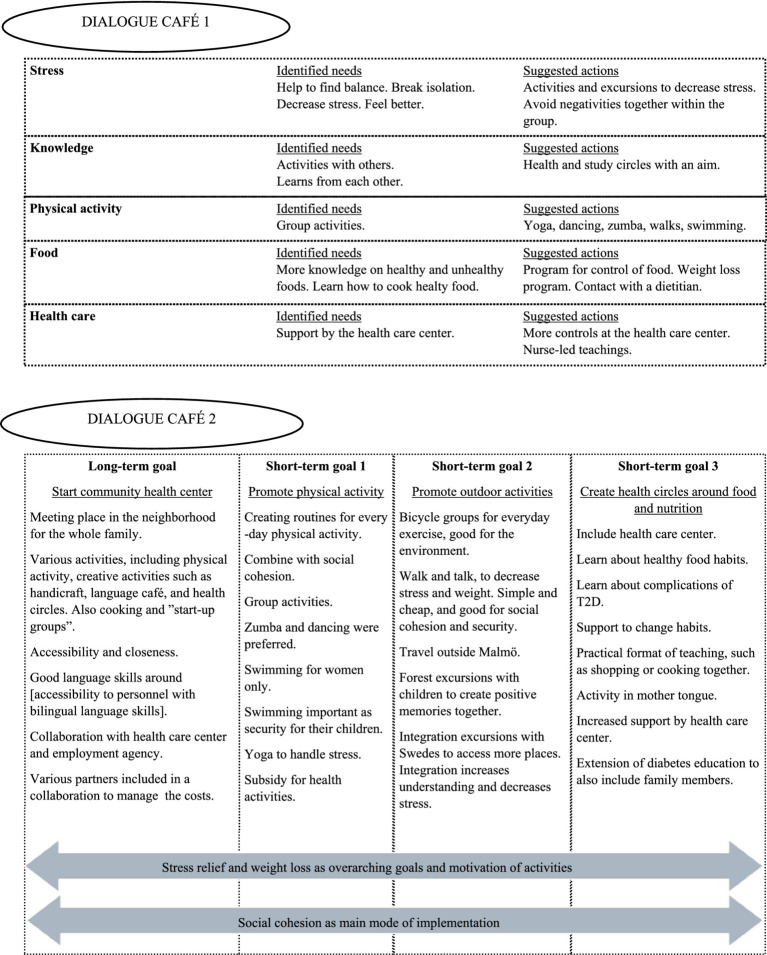
Results of the dialogue cafés 1 and 2.

Around 30 participants from the two labs accepted the invitation to the first dialogue café, resulting in six tables with around five to six participants at each table. The mind map of the answers to the third question “What do we need help with, in order to prevent the onset of diabetes?,” resulted in five themes: stress, knowledge, physical activity, food, and health care (Figure 4). Each theme consisted of identified needs and suggested actions to take to meet the needs (Figure 4).

The results from the second dialogue café showed one long-term goal and three short-term goals (Figure 4). The long-term goal was to start a community health center, and the short-term goals were to promote physical activity, promote outdoor activities, and create health circles around food and nutrition. Stress relief and weight loss were found to be the overarching goals and motivation of the activities. Moreover, social cohesion was considered important as the main mode of implementation. With those goals in mind, ideas for a CBPR intervention could start to be formed.

#### Formation of a partnership structure in relation to ideas for a CBPR intervention

It was decided that the third short-term goal—*create health circles around food and nutrition—*based upon the participatory dialogues during the second dialogue café, should be focused upon for this study. A health circle and cooperation with the local health care center was the most prioritized goal of the participants involved, since physical activity was already in place and suggested actions were improvements of those activities by, for example, including yoga and dance. Another suggestion about outdoor activities and excursions was planned to be processed later, after the winter, that is, the following year. To meet the request for more knowledge on food and food habits, a dietitian was identified in conversation with the regional health care organization, and to meet the request for more knowledge on T2D, a nurse specialized in diabetes care was identified. Both the dietitian and the nurse had participated in the second dialogue café, and both were involved in the work of the Swedish national health promotion intervention “Targeted health dialogues.” Thus, the intervention of this study was of relevance to them. A dialogue was initiated between the dietitian, the nurse, and the women, and both the dietitian and the nurse became interested in being included in the intervention facilitating team, which was a step forward toward realizing the ideas of the intervention. To meet the request for group activities, the LHPs continued as facilitators. Thus, the participatory dialogue during the planning phase, with stakeholders in the community together with representatives from the community, led to the forming of an intervention facilitating team where the women were also included (Figure 3).

#### The CBPR intervention

The intervention aimed to promote health and prevent T2D through a nurse-led education, based on reflective dialogue at the local health care center. The nurse devoted 1.5 h per week, for 10 weeks for the reflective dialogues. At the first meeting between the women and the nurse, potential themes for the forthcoming meetings were discussed. The themes they identified together were: food for persons with, or at risk of getting, diabetes; impact of physical activity on diabetes; risk factors for diabetes; feet in relation to complications of T2D; reflections around wrong information about the disease; and heredity of T2D. It was also decided to talk about hypo- and hyperglycemia as well as weight loss and stress. A more practical element of the health circle was to test the gym with a physiotherapist. Moreover, it was decided to meet at the health care center.

#### The CBPR focus group interview as qualitative evaluation tool

The first meeting with the nurse, the women said, had started with the participants sharing their needs with the nurse and then they had together decided on a theme for each meeting. Participants thought that the collaboration with the health care center was important to them, and they were grateful to all who had worked for this. It was perceived that someone listened to them and handled their questions, and they felt helped by the nurse because of her way of conducting the education. The participants also felt that they were an important part of the project because they felt listened to, and that their presence in the group was valued because they helped each other.

The CBPR focus group interview as a method for data collection engendered information about the intervention from the participants’ perspectives, as they described what factors were important to them and did not hesitate to share their experiences. For example, they stated that the fact that the nurse spoke their language helped them to have direct communication with her, which gave them the possibility to ask questions to understand more. The nurse’s dual language skills, together with her Swedish university degree, helped them to trust her recommendations.

The information also included reflections about determinants of health in relation to T2D in their community. For example, the Arabic food culture with white carbohydrates was mentioned as a reason for their T2D, as well as their physical inactivity because of their situation as migrants. War was also considered to be a reason for T2D in this community, because it leads to fear and a lot of stress, long-term stress. Moreover, the participants mentioned changed food habits, as well as being new in a country and having limited language skills. They also talked about receiving no follow-up from the health care. Thus, the focus group interview gave rise to a discussion about various reasons for the disease, implying that the group felt safe to express their opinions.

Furthermore, the participants’ accounts in the focus group interview provided information about in what way they thought the intervention helped them. They said that the intervention complemented the health care because they received education about T2D that the health care did not provide. Some had got both a diagnosis and treatment of the disease but no further education from the health care. The intervention also helped them to support each other. Besides receiving the education provided by the nurse once a week, they met each other every day, which helped them to remind each other about the knowledge they had gained. The proposal for T2D education by a nurse had come from the group themselves, but the format of the focus group could help the women involved to understand how and why this was successful.

Opinions regarding negative aspects were also shared, which indicates trust on the part of the participants in relation to the group and the researchers. For example, participants shared suggestions for improvements of the intervention after the positive accounts.

#### Modifications of the evaluation tools

In collaboration with one of the LHPs and the dietitian, a first round of modifications of the FFQ2020 was completed. A second round of modifications was completed after the cognitive group interview. Four out of five participants in the interview needed help by a translator to fill out the questionnaire. Thus, the need to design the questions/statements of the questionnaire as clearly as possible was urgent. In conclusion, all modifications could be categorized into the following themes: Irrelevant questions and responses—action: remove; Unclear relevance of questions—action: add additional response alternatives and clarifying examples; Deficient usability—action: simplify and design changes (Table 1).

**Table 1:** Modifications of the FFQ2020 and the study population demographic tool.

Examples of original formulations	Modifications	Argument for change
FFQ2020		
Sausage as fingerfood or sandwich topping (e.g., Falu sausage, German sausage, salami, beer sausage)	Remove irrelevant questions and responses. E.g., questions regarding alcohol and pork were removed as well as Swedish traditional food.	The questionnaire is too long; shortening needed.
Look at the above photos and mark the alternative that shows the portion that is the most similar to your normal portion of: meat/fish/poultry/vegetarian equivalents. A B C D If you do not eat these foods at all, choose A.	Structure the lay-out differently. Shorten and simplify the language.	The questionnaire is too complicated to go through; simplification needed.
Cookies, pastry, cake	Include examples of food relevant for the study population, e.g., mamoul, baklava, halwa.	Food used by the study population not included. Increase the relevance to the study population.
Study population demographic tool		
Where were you born? (Choose only one alternative) In Sweden In a Nordic country other than Sweden In Europe but not in a Nordic country In a country outside Europe	Skip categorization. Participants preferred to write the name of the country.	Simplification of the questionnaire needed, because categorized responses were difficult since one needs to read through all the categories to answer correctly.
Would you/your household within a month be able to pay an unexpected expense of 11,000 SEK without taking a loan or ask for help? Yes No	Remove question.	Questioned by the participants and needed to be put in a context to be understandable and answered.

Modifications of the study population demographics tool were also initiated after the cognitive group interview. Three out of five participants needed help to fill out the questionnaire demographics. Most questions were therefore either modified or removed; see example in Table 1.

The S-FHL was found to be understandable and relevant to the participants in the cognitive group interview. The first question (“Did you find the print too small to read—even if you have glasses or lenses?”) (42) was initially difficult to grasp., but after some explanation and discussion in the group it was perceived to be relevant, and it was decided to keep it as it was formulated. The questionnaire was considered to match the content of the health circle. No modifications were therefore needed.

The S-C & CHL was more difficult to understand. It was found necessary to go through all the questions first, to grasp the questions that are formulated as statements. A solution to this problem, in view of the upcoming data collection, was to make sure that the translator was well prepared and familiar with the questionnaire to be able to support the participants in responding in a valid manner. Thus, no modifications were made at this point.

## Discussion

In this study, women with a migration background living in a deprived area of a city in the south of Sweden, were collaborators in a development process for a T2D health-promotive CBPR intervention. This study describes how the exploration of the intervention development process was conducted together.

Community participants were involved from the start in dimension one as a means of managing power hierarchies, and by using a participatory approach, the participants’ knowledge about their situation, experiences and needs could be incorporated into the design of the intervention. A previous study in the program shows that women migrants experienced health care centers to be stressful and avoided asking questions regarding health issues during visits (17). However, in this study the participants accepted an invitation from the local health care center to meet at the center. Since they were meeting with the nurse as a group and not as patients, the power relations could level up and the hierarchy be broken down, thus maybe also entailing a familiarization with the health care center. This may be a way for the health care organization to build trust and it could thus contribute to integration, which Haj-Younes et al. contend can be gained by, for instance, good communication and positive experiences among migrants where this has previously been lacking (1). Because of the stressed health care system, the women did not have the power to request knowledge about diseases during normal health care visits. But when meeting in a communicative space and collecting their common accounts of the world, their testimonies could gain credibility and be communicated, and the power could be more equalized. Additionally, horizontal communication between groups of T2D patients and a health professional has previously been acknowledged as being of importance for patients’ ability of self-care (48). One part of the concept critical reflexivity is to recognize the factors influenced by power and powerlessness in one’s own situation that lead to health and ill-health (49). When the women within this study met in a participatory reflective dialogue with the nurse, they also reflected on common lifestyle factors related to diabetes. They found stress, food, and physical inactivity to be such factors and related them to migration. Which can be related to that migration is seen as a social determinant of health (50, 51).

Critical reflexivity is also about being involved in finding ways for how change can be accomplished (49). Being involved in the process and, for example, leading workshops and scrutinizing tools for evaluation, as the community were in this study, have the potential to alter the community’s ownership of the process and actions taken in the community. The CBPR approach has previously been used to develop a health promotion intervention among Syrian women in Denmark and also highlighted the importance of participation as a means for ownership of the intervention developed, something which may work to promote empowerment (52). By being involved in the process, the women in our study could share their awareness of the significance of social cohesion, and this was then acknowledged by the other stakeholders. One of the findings from the process was that group teachings were preferred. This has previously been raised within the project (17), but in this collaboration also the primary health care was involved. The use of group teachings with patients in the health care system is not new but mainly means assembling a group of patients with a shared diagnosis. To work with a group from the community means that the group might vary regarding their potential diagnosis of T2D but might instead have other things in common. Educating patients in groups about their disease is good but fits only when the included individuals already have contracted the disease. It is a tertiary prevention strategy (10), and thus not a health promoting approach. However, both strategies are important and complement each other (10). Usually, in health research that is not participatory, the aim is be to improve the health of the general population or of a patient group, whereas CBPR and PAR in health research are more concerned with enabling people to act themselves, through critical reflexivity (49). Even though not all of them had diabetes, they all perceived the education as useful, partly because some had relatives with diabetes and partly because the education had a general health-promotive approach. It may not be cost-effective for the primary health care center to teach the whole population in a community about diabetes but migrants from the Middle East have been identified as a risk group for contracting T2D (4). As suggested by Nutbeam, interventions to target C & CHL among community populations are needed (27). The way of working suggested in this study is opposite to the health care visit where the health care professionals usually meet individually with patients. This may therefore be one way to address C & CHL where the community is in focus.

The result shows that the participants within the group supported each other. To engage the community in interventions has previously been shown to be useful to change health behavior and promote social support (53). It can affect the process of community empowerment, and not only the individual, by building community capacity, something that may potentially lead to more social justice, which could, ultimately, reduce health inequities (53). Promoting better health within the whole group in a health promotion program with reflective participatory dialogues, can build up community capacity collectively and thereby reduce the sole reliance on patients’ individual self-care. Additionally, a group from the community, as in this study, may have a more extended agenda to meet than the official activities organized, and this may increase the social support. This is what the participants joining the intervention said, namely, that they meet every day and therefore can support each other in between the teachings with the nurse. Thus, peer support may be stronger in an already existing group based in a community, and it may be more effective to use that group for education even though not all are patients, as it may extend the potential success of the teachings offered by the health care center and, additionally provide sustainability. So, even though it may be more costly for the primary health care center to conduct education with others than only patients it may be more effective since they help each other within the group so that the knowledge sustains. And also, because they help people in their surrounding having diabetes.

The cognitive interviews resulted in suggestions for improvements of the evaluation tools. It also showed that some of the participants needed help to fill out the questionnaires. As the LHP was translating and helping out as needed to do so, it resulted in that all participants could take part in the scrutinization of the evaluation tools. This was important because everyone’s knowledge could then be considered. Thus, one can think that to include this population extra work may be needed, but which may also be of extra importance. For example, it has previously been mentioned that this population has been excluded from the public health questionnaire in Sweden, because of language limitations (54). And even though the health literacy questionnaires were short and therefore considered appropriate, there was still a need to discuss and put the questions in a context to be able to answer the items, which was also elucidated in the dialogues.

It has been shown that to succeed in preventing T2D, multiple interventions should be ongoing in combination and on various levels, including the individual, group, and societal level (55). As it happened in this study, both the nurse and the dietitian collaborated in the local intervention as well as working for the national intervention “Targeted health dialogues.” The purpose of the national intervention is to screen all 40–50-year-old individuals in order to identify unhealthy lifestyle habits and give support for changes to prevent T2D and cardiovascular diseases (56). The nurse met individually with people from the community in the health care center for screening, whereas the dietitian worked on the regional and national strategic level of that intervention. Together, they covered all three levels, which makes for a better chance of success for all interventions. Coincidentally, the CBPR program was integrated into already existing structures while also including a bottom-up perspective. This study does also describe how an intervention could be developed in collaboration in relation to a clinical setting. Due to the participatory approach of this study, and thus the collaborative management, the need to involve a nurse specialized in diabetes care in the intervention, could be met. In a systematic review of health literacy interventions for migrant populations, it was found that nurses did not take part in interventions, even though nurses have a health-promoting position and should therefore be prone to deliver such health initiatives (57). Unfamiliarity with health literacy and uncertainty about how to assess it in patients were discussed as possible reasons for this (57). However, interventions aiming to increase health literacy among the migrant population may also increase the understanding among the health care personnel providing the intervention (58). Furthermore, another reason why nurses do not take part in such interventions may be lack of time (25). The new reform in Sweden called “Nära vård” (‘Close care’), where the primary health care is supposed to conduct health-promoting work, may be difficult to accomplish within the current health care organization with its already limited resources for more acute health care (25). The health-promoting approach and salutogenic thinking have usually not been given enough priority within the health care organization, which is more focused on preventing or treating diseases (10). Nationally, the government and the Swedish Public Health Agency are responsible for health promotion (10), but locally there is usually no specific place or organization where an individual can seek help to promote their own health building capacity in the local communities. Instead, the responsibility for the health promotion work is usually on an individual level. Sweden has policies on a national level to promote social determinants of health, and equal health care is financed by the public sector, but there is no strategic local governance structure that includes local communities in the decision-making of self-care. Instead, a governmental structure tends to create silos in the system that burden the primary care units further (30). New ways of working are thus suggested (25). The health care organization does not alone have the responsibility for the health-promotive work; however, the primary health care is a given stakeholder for initiating such work locally, together with other stakeholders, which may free resources within the health care to be used for others (25).

### Strengths and limitations

One strength of this study is the facilitation of communicative space to enable dialogue. The communicative space environment is important to pay attention to, by choosing the right place and making sure that the people involved work with power and hierarchies. The time and effort invested in this is rewarded in the shape of trust and, not least, in the knowledge resulting from the process.

A limitation of this study was that not all members of the hub could be involved in every step of the process. However, this is not necessary within participatory research (36), and all have contributed their skills and been involved in deciding who is doing what.

### Ethical discussion

As issues of power are known to be ethical challenges in collaborations in CBPR (47), this is something the research group has worked with and striven to increase awareness of. To give the community members interpretive priority is one example of that work in this study. Cultivation of communication based on humility and an absence of prestige is another example. A further ethical challenge in participatory research is that partners do not always have the same expectations or timelines (47). This was experienced also in this study, and the researchers and the LHPs tried to manage this challenge through transparent communication and by spreading information regarding the steps of the development process to the community and other stakeholders involved in the process. To communicate to the participants why it took time for the intervention to start, for example, felt essential for the relationship of trust.

## Conclusion

The process that has been explored in this study highlights how participants’ knowledge can be used in the development of a T2D health-promotive CBPR intervention among women with a migration background. The conceptual CBPR intervention model meant that women from the community were collaborators already from the start of the process, which was crucial in aiming for shared power between the stakeholders. Thus, active work to equalize power permeated the development process, by the use of methods enabling people to be involved. Through using the dynamic of reflection and action in cooperation, the needs of the community could—in dialogue—be met by other stakeholders and a joint intervention and evaluation could be developed. There may be potential to build groups for T2D health promotion based on community instead of patient category and thereby benefit from an already existing supporting group in working for an even stronger effect of an intervention. But this needs to be further researched. Primary health care needs new ways of working in a health-promotive way for the population and especially for groups such as those described in this study. The nurse in primary health care is in a strategic position to bridge the gap between the health care organization and the local population and build health-promotive communities. Lack of time and lack of a salutogenic tradition within the health care organization may hinder nurses from managing such tasks, however. The CBPR approach offers one way to deal with that issue.

## Data Availability

The datasets presented in this article are not readily available because the data generated and/or analyzed during the current study are not publicly available due to GDPR and secrecy but are available from the corresponding author on reasonable request. Requests to access the datasets should be directed to margareta.ramgard@mau.se.
