# Erratum: The development process of a type 2 diabetes health-promoting CBPR intervention

**DOI:** 10.3389/fpubh.2025.1576910

**Published:** 2025-02-27

**Authors:** Frontiers Production Office

**Affiliations:** Frontiers Media SA, Lausanne, Switzerland

**Keywords:** type 2 diabetes, health literacy, peer support, community-based participatory research, migration, women, health promotion

Due to a production error, the affiliations for Cecilia Lindsjö, Katarina Sjögren Forss, Christine Kumlien, Anders Kottorp, and Margareta Rämgård were incorrect. Instead of:

“Cecilia Lindsjö^1*^, Katarina Sjögren Forss^1^, Christine Kumlien^2^, Anders Kottorp^1^ and Margareta Rämgård^1^

^1^Research centre Promotion for citizen Health, Malmö University, Malmö, Sweden

^2^Department of Care Science, Faculty of Health and Society, Malmö University, Malmö, Sweden”

It should be:

“Cecilia Lindsjö^1, 2*^, Katarina Sjögren Forss^1, 2^, Christine Kumlien^1^, Anders Kottorp^1, 2^ and Margareta Rämgård^1, 2^

^1^Department of Care Science, Faculty of Health and Society, Malmö University, Malmö, Sweden

^2^Research Centre Promotion for citizen health, Malmö University, Malmö, Sweden”

Due to a production error, there was a mistake in Figure 2 as published. The word for product manager was written incorrectly and not updated as requested. The corrected Figure 2 appears below.

**Figure 2:**
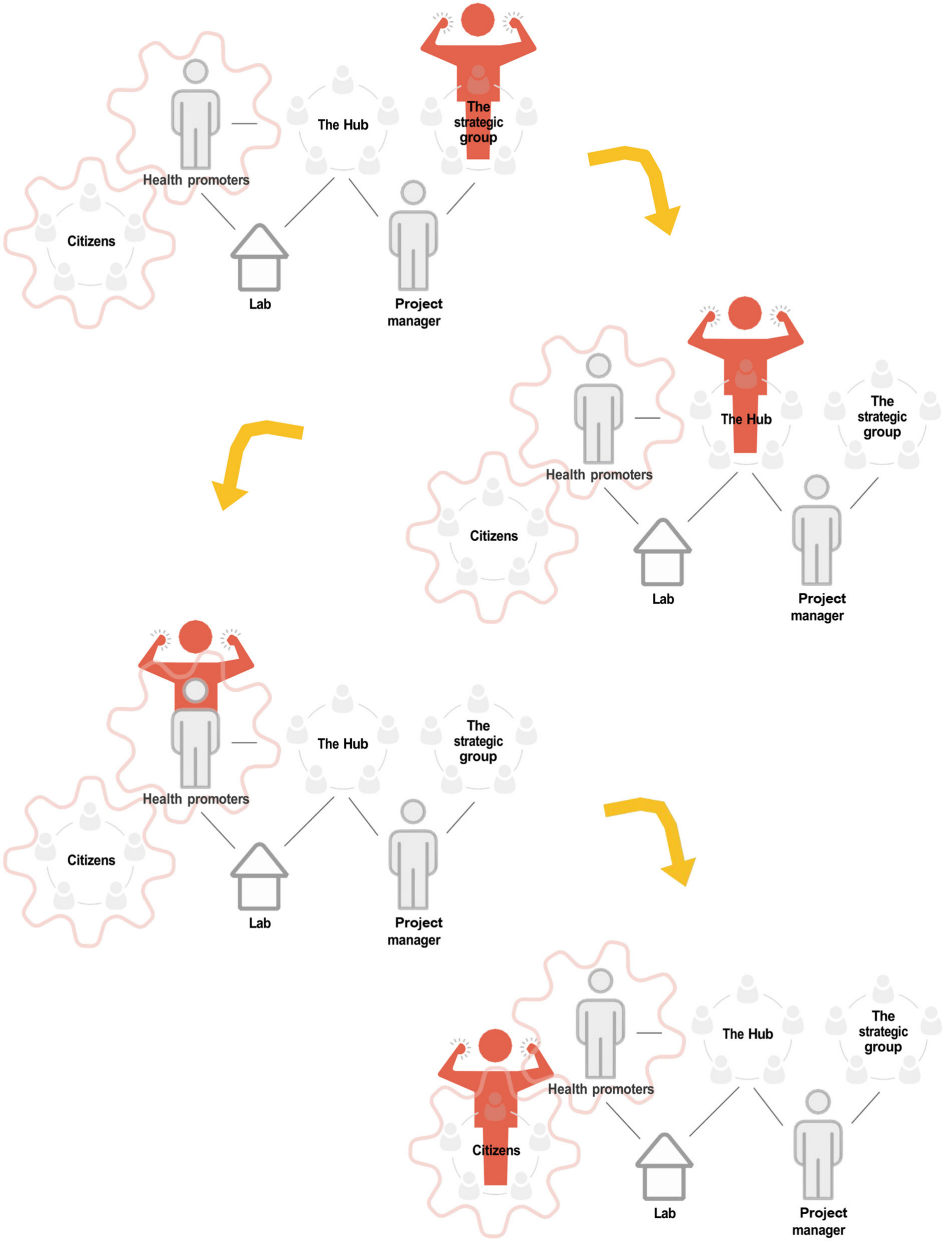
Working model in the collaborative innovations for health promotion program of how power should be transferred (32).

The publisher apologizes for this mistake. The original version of this article has been updated.

